# The care burden for technology-dependent children with long-term home ventilation increases along with the improvement of their motor functions

**DOI:** 10.1007/s00431-023-05249-w

**Published:** 2023-10-16

**Authors:** Hirotoshi Maeda, Ikuko Tomomatsu, Izumi Iikura, Masahiro Ikari, Youichi Kondo, Miyuki Yamamoto, Masanori Tamura

**Affiliations:** 1 Medical Incorporated Foundation Harutaka Kai, Ueno Tosei Bldg. 9F, Higashi-Ueno 4-23-7, Taito-ku, Tokyo, 110-0015 Japan; 2 TOMO Lab LLC, Shibuya-ku, Tokyo Japan; 3 Graduate School of Human Sciences, Osaka University, Suita-city, Osaka Japan; 4 Institude of Human Sciences, University of Tsukuba, Tsukuba-city, Ibaraki Japan; 5 Faculty of Medicine, Saitama Medical University, Saitama-city, Saitama Japan

**Keywords:** Technology-dependent children, Care time, Self-rated care burden, Pediatric home medical care, Ventilation, Tracheostomy

## Abstract

Since the establishment of Japan’s publicly supported pediatric home medical care (PHMC) system in 2012, the number of technology-dependent children (TDC) who are supported by PHMC has been increasing. We expected care to become easier as TDC mobility increased. However, many family caregivers complained of the opposite, that the burden of care increased as TDC’s mobility increased. Therefore, we aimed to study the problems of the care burden of families in relation to the improvement of mobility of TDC with a focus on respiratory support. We conducted a survey among caregivers of TDC in two parts: a first survey was done between 2018 and 2020, and a follow-up survey in 2023. In each medical procedure, the correlation of TDC’s motor function level with care burden was analyzed by using correlation analysis. In 15 households, caregivers’ activities were monitored to elucidate the problems of the care objectively. The second survey was conducted in February 2023 in TDC whose medical records were available. Analysis of 418 cases showed that care-burden increased with improvements in the children’s motor ability in tracheostomy, or remained the same in other procedures. A follow-up study in 262/418 cases of this group showed that part of these TDC with high mobility aged 1–8, after passing through the mobile phase with increased burden of care, had a high potential to be weaned from medical devices.

*Conclusion*: For TDC who acquire motor skills during PHMC, the burden of care for caregivers either increases or remains the same. It is important to maintain social support for TDC on the recovering mobile stage.

**Table:** 

**What is Known:** *• There was no clear information on the care burden when TDC become mobile.*	
**What is New:** *• We did a qualitative study to show that the care burden increases when TDC become mobile.* *• In addition, mobike TDC showed a high potential for recovery.*

**Supplementary Information:**

The online version contains supplementary material available at 10.1007/s00431-023-05249-w.

## Introduction

Since the first definition of Children with Medical Complexity (CMC) was proposed by Cohen and colleagues in 2011 [1], their number seems to be increasing worldwide [2–4]. As a subcategory of CMC, the term *Technology-Dependent Children* (TDC) [5] has been used to refer to children who continuously need clinical technology such as long-term ventilation to support their biological functioning. The increase in TDC numbers resulted in the need for community shifting of these children from hospitals [6, 7], and health and social systems for pediatric home medical care (PHMC) have been built in various countries. In Europe, multidisciplinary teams (MDT) are involved in the care of TDC; however, team compositions vary depending on nations from the level of individual professions being in charge of a patient, hospital MDT, partially mixed MDT, to fully-mixed MDT [8]. In the USA [9] and in Canada [10] as well, structures of MDT vary depending on areas, states, or types of health insurance.

**Fig. 1:**
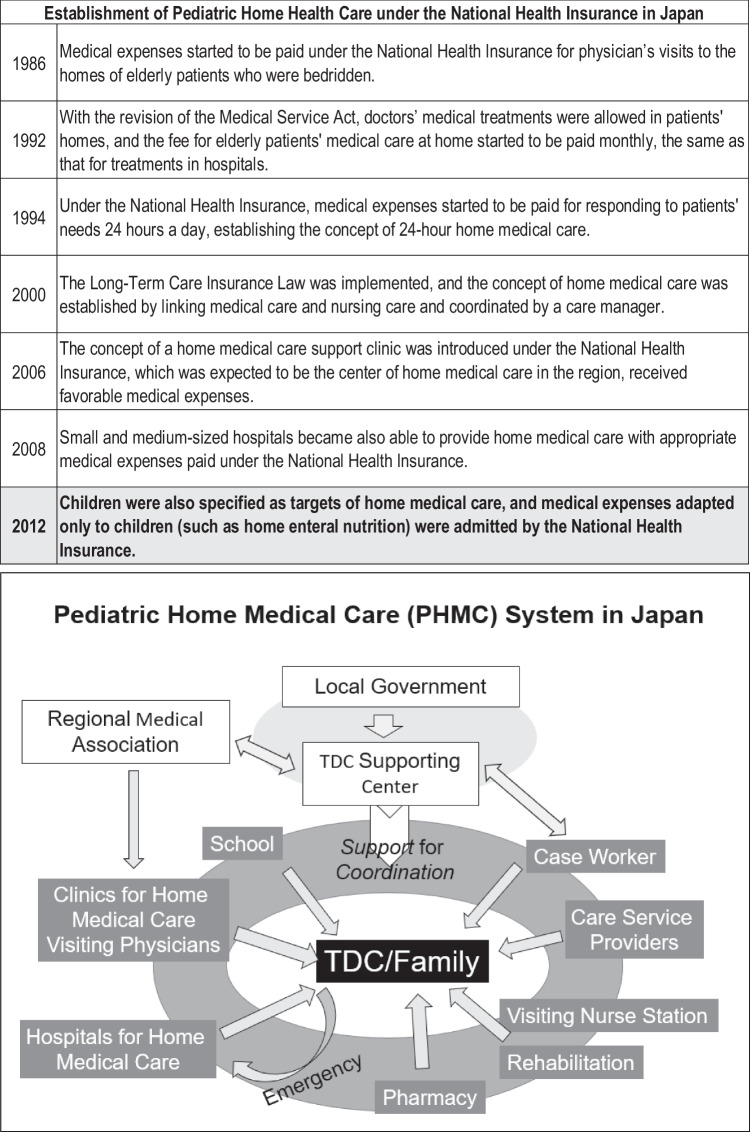
History of Pediatric home medical care (PHMC) in Japan (top), and the diagram of the current PHMC system in Japan. Note that, in the Japanese system, physicians visit patients’ homes on a regular basis

In Japan as well, the lifesaving rate of neonates has dramatically improved, resulting in a rapid increase in the number of children who need technological support in everyday life [11], but available NICU beds became fully occupied. It, therefore, became necessary to promote shifting their care to the community. As institutions that take care of such children were also already fully occupied with a heavy care burden [12], treating TDC at home became an urgent need. A home-visit medical system, covered by national health insurance, had already been started in the late 1980s in Japan. However, the system was limited to patients 65 years and older and was not available for children. To meet the urgent demand, a PHMC system was established around 2011–2012 by the government (Fig. 1). What is noteworthy about this Japanese system was that physicians visit patients’ homes and treat them there. In this system, support for PHMC is provided by both the national health insurance system and the social welfare system, including long-term care insurance and programs for severely disabled children. There is inter-professional cooperation among various professions. Local governments have coordinated a system of visiting physicians, visiting nurses, social workers, pharmacists, and home helpers who provide support for household work, etc. This has made it possible for TDC to be cared for in their own homes. In the most severe cases, patients die at hospitals, but TDC who attain a stable state in their physical condition are discharged from hospitals in less than 1 year and are assigned a PHMC-providing clinic when their parents decide to take them home.

As the number of TDC who receive PHMC service kept increasing, TDC who can “move voluntarily” but need medical devices for 24 h also increased. Then a problem became apparent. The general consensus at the beginning was that care would become easier as the TDC’s motor function improved. However, on the contrary, many caregivers complain that their burden increased along with the increase in TDC’s mobility. This led us to survey the care burden in relation to the improvement of TDC’s health conditions including motor function to provide data validating a request to change the public social support system for TDC. In this study, we first focused on how caregivers’ care burden differed depending on TDC’s motor function, then with their follow-up study, we discussed the potential and needs of these children.

## Methods

### Questionnaire survey of care time and Self-rated Care Burden (SCB)

We conducted surveys in 2 time points. For the first stage of the survey, the customed questionnaire was sent to 1162 TDC households with the cooperation of 18 medical institutions in Japan by post mail between May 2019 and February 2020. After receiving 552 answers (47.5%), we analyzed 418 cases (36.0%) of children aged 19 and under.

As shown in Table 1, caregivers provided information about the TDC’s age, gender, their main caregivers, family structure, devices they use, and motor function level (from level 1 to level 8: according to the commonly used criteria in Japan [13, 14]). TDC’s health conditions were categorized from the medical records of 262 TDC out of 418 (Table 2). We classified medical procedures into 12 categories as shown in Table 1, and each procedure was further divided into its subcategories. To fill in the questionnaire, family caregivers selected a category of medical care they provide from the list (Table 1) and then its subcategories, and assessed the Self-rated Care Burden (SCB) level for each subcategory on a 5-point scale as shown in Table 1. The time spent for each procedure was entered into the time column on the sheet. Caregivers also recorded the starting time of each procedure, from which we calculated the frequency with which the procedure was implemented. Care time (CT) for each subcategory was calculated as follows: *representative CT for each medical procedure* × *its frequency/24 h*. The total CT of each category was the sum of the CTs of its subcategories. For calculating the SCB of each category, first, the *SCB of each subcategory* × *frequency/24 h* was calculated as the *subcategory SCB score*, and then all *subcategory SCB scores* were added to arrive at the *SCB* score for the category in question. For statistical analysis, the correlation analysis by Pearson was used. Correlation analyses between care time vs. SCB, and that between levels of motor ability (1 to 8) vs. CT, or vs. SCB were performed for each category of medical care.

**Table 1:** Main questionnaire items

**Categories of medical care**	
1	Tracheostomy
2	Suction
3	Ventilator
4	Mechanical exsufflation
5	Continuous suction
6	Airway insertion
7	Nebulizer
8	Oxygen therapy
9	Tube feeding (Gastrostomy, Intestinal fistula, Nasal and oral gastric feeding tubes, Nasal Elemental Diet tubes)
10	Intravenous Hyperalimentation (IVH)
11	Peritoneal Dialysis
12	Self-urinary catheterization

**Motor function levels 1–8**	
Level	Motor Ability
1*	no voluntary movement
2*	able to move one’s neck
3*	able to move one’s upper and/or lower limbs
4*	able to move one’s trunk
5*	able to sit up
6	able to crawl
7	able to stand up
8	able to walk

**Self-rated Care Burden (SCB) score 0–4**	
score	Evaluation Criteria
0	I don’t feel any burden at all
1	I feel slightly burdened
2	I feel fairly burdened
3	I feel heavily burdened
4	I feel extremely heavily burdened

*Japanese welfare support system for children with severe motor and intellectual disabilities (C-SMID) covers children up to level 5

**Table 2:** Demography of TDC

**Region**	**Cases**	**%**
Tokyo	236	56.5
Kanto	76	18.2
Hokkaido	37	8.9
Kinki	20	4.8
Kyusyu	19	4.5
Tohoku	11	2.6
Hokuriku	10	2.4
Tokai	9	2.2
Total	418	100.0

**Sex**		
Male	196	46.9
Female	180	43.1
No answer/unknown	42	10.0
Total	418	100.0

**Family structure**		
Mother only	16	3.8
Mother+siblings	12	2.9
Mother+grandparents	4	1.0
Father only	0	0.0
Parents	141	33.7
Parents+siblings	194	46.4
Parents+grandparents	12	2.9
Parents+siblings+grandparents	17	4.1
Other	22	5.3
Total	418	100.0

**Main caregivers**	**Cases**	**%**
Mother	398	95.2
Father	8	1.9
Parents	9	2.2
Parents + other	1	0.2
No answer/unknown	2	0.5
Total	418	100.0

**Disease categories**		
Perinatal disorder (brain damage +)	52	12.4
Perinatal disorder (brain damage -)	5	1.2
Neuromuscular disease	50	12.0
Multiple congenital malformation syndrome	45	10.8
Chromosomal abnormality	35	8.4
Internal organ disease	30	7.2
Postnatal disorder (brain damage +)	24	5.7
Metabolic disease	12	2.9
Tumor	9	2.2
Unknown	156	37.3
Total	418	100.0

**Devices**		
(1) Tracheostomy only	15	3.6
(2) Ventilator only (NIV)	24	5.7
(3) Gastrostomy tube feeding only	45	10.8
(1)+(2) (IV)	24	5.7
(1)+(3)	38	9.1
(2)+(3) (NIV)	73	17.5
(1)+(2)+(3) (IV)	176	42.1
Other than the above	23	5.5
Total	418	100.0

*NIV* non-invasive ventilation, *IV* invasive ventilation (with tracheostomy)

### Study with time-lapse camera

We recruited 15 TDC (7 TDC for the periods from December 2018 to January 2019, and 8 TDC from February to June 2020). For each household, up to 8 cameras (Sopak-C, Kobayashi Manufacture) were set up for fixed-point observation. Camera recording was conducted 24 h/day, at 5-min intervals (288 frames), but the recording time varied from 5 to 24 h depending on each family’s convenience. Afterward, each time frame was manually marked by care type and the total CT was calculated for each child. Children’s behavior in response to medical devices used was also recorded.

### Follow-up study

The second stage of the survey was conducted in February 2023 to follow the outcome of TDC who gained motor function. Among the 418 cases initially included, 262 TDC (63%) had available medical records and in these 262 cases, we analyzed weaning rates from their devices in relation to the categories of their diseases (Supplementary Table 1) and devices in use. For statistical analysis, the Chi-square test was used.

#### Ethics approval

This study has been approved by the Ethics Committee of Harutaka-kai (Approval Number 2018_3, Approval Date 05/30, 2018) and the Ethics Committee of Saitama Medical University (Approval number 2219/ Approval Date 12/26/2019). This study was performed in line with the principle of the Declaration of Helsinki.

#### Consent to participate

Written informed consent was obtained from the families of TDC for approval Date the questionnaire survey and the filming of their care activities in their homes.

## Results

### Self-rated questionnaire survey

Demographical profiles of the valid 418 cases of TDC are summarized in Table 2.

#### Region

The survey area included almost all regions of Japan with the highest number in Tokyo. As the regional distribution pattern of TDC is similar to that of clinics providing home medical care in Japan, we surmise that the results reflect trends across Japan.

#### Sex

The numbers of male and female TDC are almost equal.

#### Family structure and main caregiver

Various combinations were observed with parents or parents and siblings the most common combination. In terms of the main caregiver, 95% were the mothers. One notable feature was that the ratio of single parents was 7% (cf. 1.24% in general households in Japan in 2019), and that while there were cases of the *mother only*, there were no cases of the *father only*. The main caregivers were mothers in 398/418 cases (95.2%).

#### Disease categories

Medical conditions of TDC whose medical records were accessible (262/418) were classified into 8 categories (Table 2) (for disease names in each category, refer to the Supplementary Table 1). Perinatal disorders account for the largest number, followed by neuromuscular disease, multiple congenital malformations, chromosomal abnormalities, and internal organ diseases. Numbers in () indicate TDC who died during the survey period (2018–2023).

#### Devices

In terms of 3 major devices (tracheostomy+ventilator+gastrostomy tube feeding), 20% of TDC needed only a single device, 32% needed two devices, and 42% needed 3 devices. The result indicates that TDC with a wide variety of diseases in severe conditions who need multiple-device support are covered by PHMC.

#### Age

**Fig. 2:**
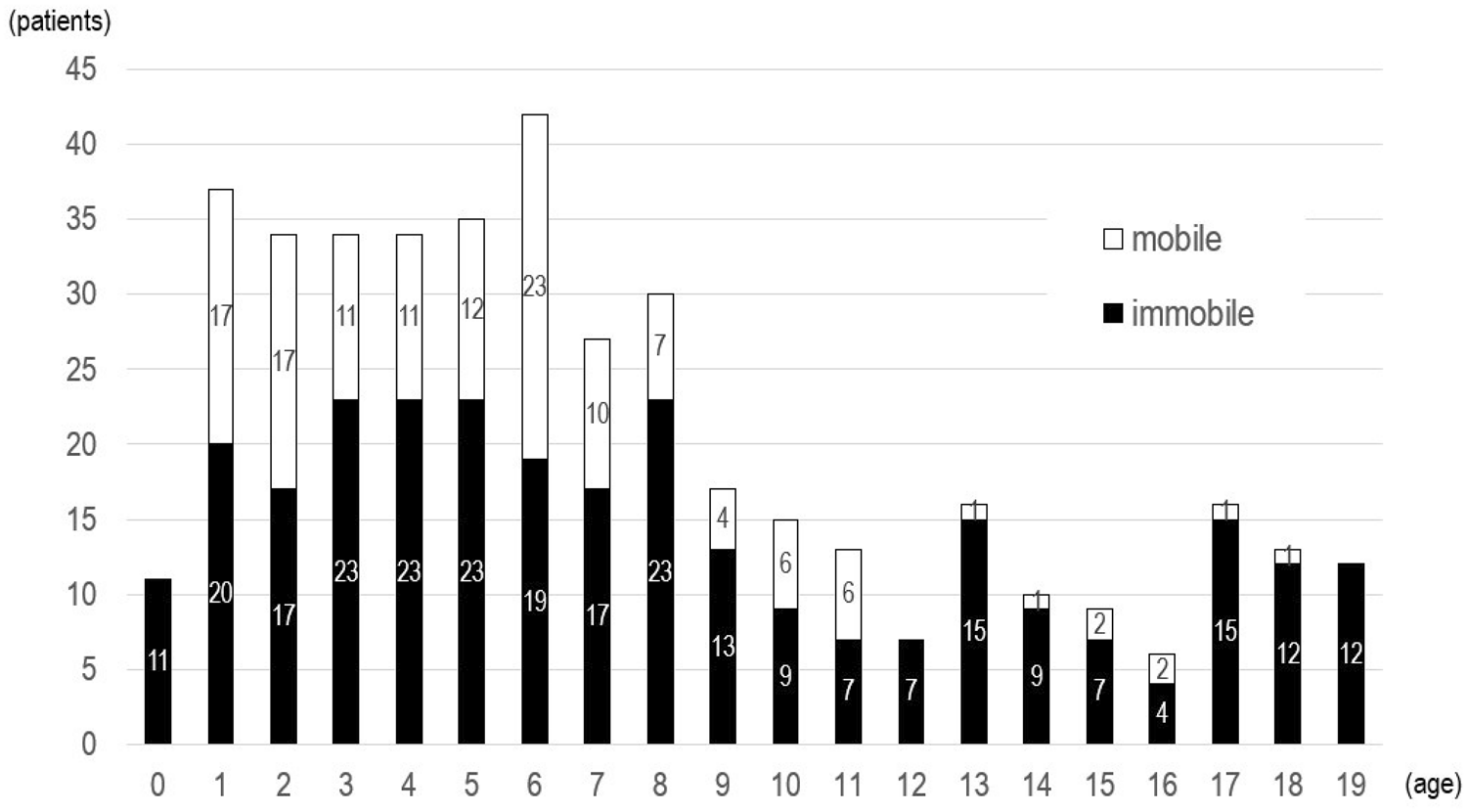
Age distribution of surveyed TDC with the level of motor function. Numbers in the axis indicate the number of patients. Abscissa is the age of TDC. Filled columns indicate the number of immobile (Levels 1–5) TDC and white columns indicate that of mobile (Levels 6–8) TDC

The age distribution of TDCs together with the level of their motor function is shown in Figure 2.

The number of TDC under 1 year of age is low because many of this group are still in hospitals. The high numbers were observed in the 1-to-8-year-old group among both mobile (Lv.6-8) and immobile (Lv.1-5) TDC. In this age group, the ratio of mobile vs. immobile TDC was almost 1:1 (age 1, 2, 6) or 1:2 (age 3, 4, 5, 7). However, above the age of 9, the number of both groups decreased, and above the age of 12, the decrease was more prominent in the mobile group.

### Care time (CT) and Self-rated Care Burden (SCB) in relation to the level of TDC’s motor function

**Fig. 3:**
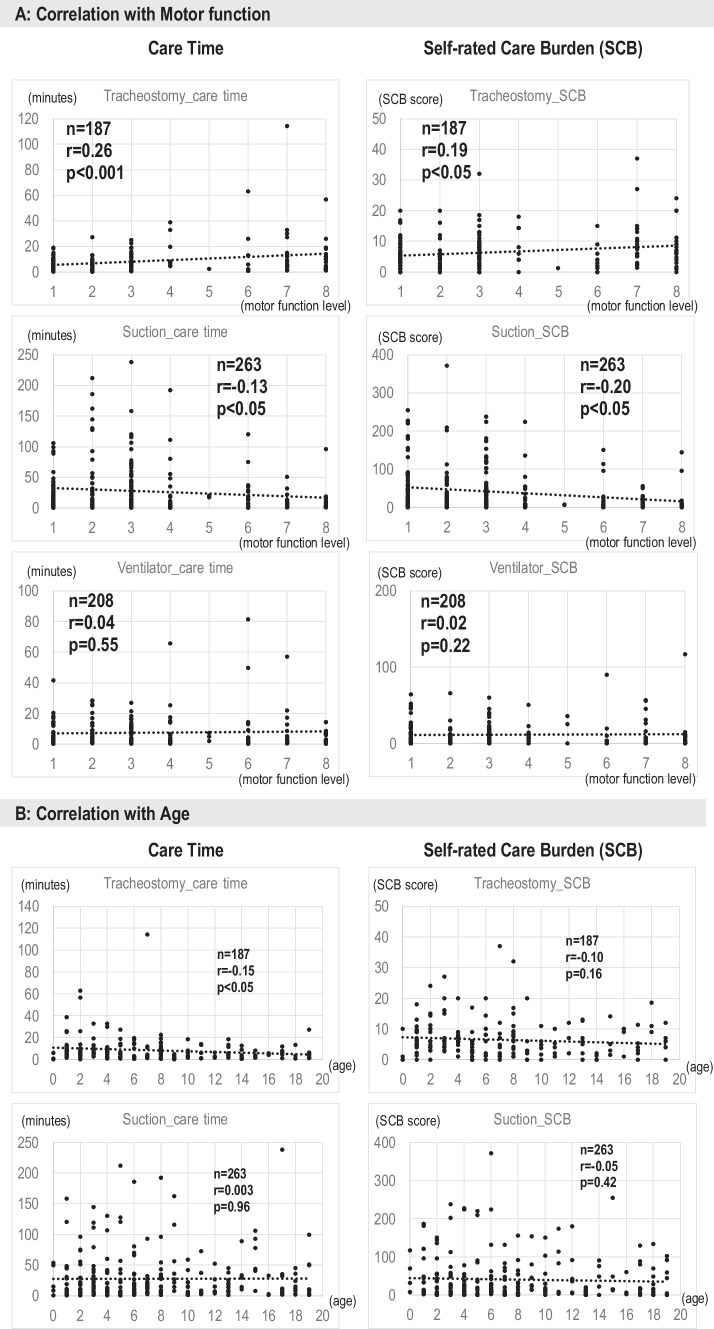
**A** Correlation between motor function level (1–8 abscissa) and CT (left column, in minutes), and between motor function level and SCB (right column, in SCB scores) with 3 devices. **B** Correlation between age (abscissa) and CT (left column), and between age and SCB (right column)

We analyzed the level of care burden in the 418 cases by using indices of the total CT and SCB scores, and how those scores were affected by the child’s level of motor function (Level 1–8). For this analysis, medical procedures used by a population of less than 100 were excluded from the analysis, and we focused on 4 categories: *tracheostomy*, *ventilator*, *suction*, *and gastrostomy tube feedings*. First, we analyzed whether CT and SCB change proportionally, and all 4 procedures showed a moderate correlation between CT and SCB (*r* = 0.56, 0.4, 0.53, 0.47 respectively with *p* < 0.001 for all). Correlation analysis of CT or SCB with motor function level (1–8) showed a significant positive correlation in tracheostomy, both in CT and SCB. This indicates that the burden of care related to the tracheostomy increases as the child is more mobile. Neither ventilators nor gastrostomy tube feeding showed statistically significant correlations, indicating that the level of care burden for these procedures remains the same regardless of TDC’s motor function level (Fig. 3). Only in suction, did both CT and SCB show a negative correlation (Fig. 3), indicating that the care burden for this procedure decreased as the child’s motor function improved. TDC who cannot move their bodies always take a lying position that obstructs their air tract due to the difficulty of expelling phlegm and requires frequent suctioning, whereas mobile children are more likely to expel the phlegm on their own. The overall result showed that as TDC’s motor function improves, the care burden either increases or stays the same except for suction.

### Age is not related to care burden

As TDC grow in age, their physical abilities increase. Therefore, we examined whether the positive correlation between tracheostomy and motor function, or the negative correlation with suction, is related to their age. As shown in Fig. 3, the level of care burden with these procedures did not change in relation to TDC’s age.

### Study with time-lapse camera

To verify the questionnaire survey, we then analyzed the relationship between motor function level and CT objectively by using a time-lapse camera. We also attempted to find other factors of care burden that the questionnaire survey did not reveal. The CT varied from 16 to 48% of the total recording time. The CT and the motor function level did not show a significant correlation which may be due to a lack of power (small sample). The analysis of the time-lapse camera did reveal, however, that TDC with high motor function frequently removed their ventilator catheter mount by themselves and caregivers had to reattach it each time; therefore, we counted the frequency of removing the catheter mount with 4 subjects who used the same devices and had the same time frame of recording but in different motor function level (Table 3). Child #4, with motor function level 8, removed the catheter mount 27 times in 7.5 h, while the other 3 children with levels 1–3 removed it none or only 4 times. This observation showed that some TDC with high motor function need constant attention, which places a heavier burden on caregivers.

**Table 3:** List of video-monitored TDC

ID	Sex	Age of the survey	Motor function level	CareTime(min)	Recording time	CareTime /RecTime(%)	Frequency of removing catheter mount (9:55–17:25)	Devices		
								Tracheostomy	Ventilator	Gastrostomy
1	Male	3	1	485	24h	33.68%	0	1	1	1
2	Female	2	2	690	24h	47.92%	–	1	1	0
3	Female	8	2	450	24h	31.25%	–	0	1	1
4	Male	4	2	550	22h	41.67%	–	1	0	1
5	Male	6	3	690	24h	47.92%	0	1	1	1
6	Female	2	3	630	24h	43.75%	4	1	1	1
7	Male	2	3	440	16h30m	44.44%	–	0	1	1
8	Female	1	5	240	8h30m	47.06%	–	1	1	1
9	Female	6	6	150	5h30m	45.45%	–	1	1	1
10	Female	4	6	85	5h10m	27.42%	–	0	0	1
11	Male	3	7	50	5h10m	16.13%	–	1	1	0
12	Female	4	7	305	10h	50.83%	–	1	1	0
13	Male	1	7	120	7h30m	26.67%	–	1	1	0
14	Female	11	7	145	9h	26.85%	–	1	1	1
15	Female	3	8	175	7h30m	38.89%	27	1	1	1

### Follow-up study of TDC

After this survey (2018–2020), in February 2023, we examined 262 cases (out of the 418 surveyed cases) in which we had access to their medical records. The total mortality rate during 4.5 years (2018–2023) was 24/262 = 9.2%, with chromosomal abnormality (5), perinatal brain damage (5), multiple congenital malformation syndrome (5), and tumor (4) the major causes of death.

In the questionnaire survey conducted on 2019–2020, TDCs with tracheostomy accounted for 253/418 = 60.5%, and its care burden increased with improved motor function. With ventilators, which accounted for 297/418 = 73.4%, behavioral troubles also increased with improved TDC motor function. Therefore, in the follow-up study conducted in 2023, we also focused on tracheostomy and ventilators. In 262 TDC in the follow-up study, 149 (56.9%) had tracheostomy, 203(77.4%) had ventilators (both IV and NIV), and 124 had both devices (34 cases did neither have ventilator nor tracheostomy, but were attached with other types of devices such as stomach tube feeding).

**Fig. 4:**
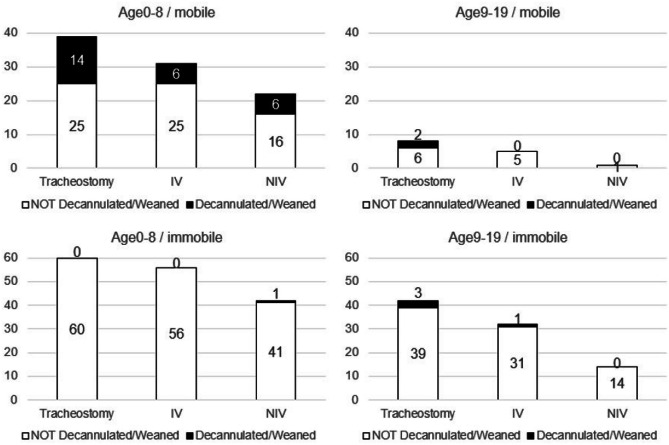
Age- and mobility-dependent withdrawal cases from tracheostomy and/or ventilator. TDC who are “mobile” are Lv.6–8 and not supported by C-SMID, and “immobile” indicates those who are Lv.1–5 and cannot move on their own (see also Table 2). The axis shows the number of cases. Black columns indicate cases that were decannulated or weaned from the ventilator, and white columns indicate cases that continuously need the devices at the time point of February 2023. In this figure, TDC with multiple devices are counted in both device categories

In Fig. 4, the numbers of cases that were decannulated or weaned from ventilators over the period from December 2018 to February 2023 were shown. In this figure, a case that needed multiple devices was counted in both device categories (therefore the total exceeds 262). In the 0–8 age group, mobile TDC (Lv.6–8) showed the highest decannulation rate (35%) and weaning rate from both invasive ventilation (IV) (19%) and non-IV (NIV) (27%), while it was 0–0.02% in the immobile group (Lv.1–5). The result indicates that mobile TDC with tracheostomy and/or ventilator have more potential to be decannulated or weaned from the ventilator in this age group. The decreased number (and ratio) of mobile TDC in ages 9–19 may indicate that many of them could leave PHMC around age 9. This observation could be explained that TDC who were decannulated or weaned from a ventilator had symptoms of tracheomalacia or airway stenosis, and these symptoms improved as they developed. For statistical analysis, we grouped TDC with ventilator and/or tracheostomy, and compared withdrawal rates from these devices in 4 groups: age 0–8 immobile, age 0–8 mobile, age 9–19 immobile, age 9–19 mobile. The chi-square test showed that the withdrawal rate from devices was significantly higher in the group of age 0–8 mobile than in other groups (χ^2^ (3, *n* = 228) = 41.64, *p* < 0.001). Thus, the percentage of TDC who could leave devices was higher in the mobile group than in the immobile group, and factors or characteristics of groups of TDC who could leave devices need to be analyzed in future studies.

In conclusion, this follow-up study indicated that children who became mobile were those most likely to be weaned off their devices.

## Discussion

### Improvement of motor function increases the care burden

In Japan, the total number of TDC who use a ventilator at home has been increasing, exceeding 5000 in 2021 [11]. Almost half of these children use Invasive Ventilation (IV) with tracheostomy and this ratio has remained unchanged for the past 10 years. In our survey of 418 TDC as well, about half (47.8%) required IV, while those using Non-Invasive Ventilation (NIV) was only 21.7% (Table 3). High IV number is a distinguishing characteristic of Japanese PHMC services as compared to other countries where the NIV number is increasing at a more rapid pace [15, 16]. However, recent reports indicated that tracheostomy is beneficial for the development of children who have pulmonary or cardiac symptoms [17, 18], and with careful monitoring, their conditions can be well maintained [19]. Japanese PHMC standards of care for respiratory disease are consistent with those of “an official American Thoracic Society clinical practice guideline: pediatric chronic home invasive ventilation [20].

TDC with tracheostomy accounted for almost half of the 418 cases and the care burden for tracheostomy increased as TDC’s motor function improved (Fig. 3). In addition, in other medical procedures, except for suction, the care burden remained at the same level irrespective of TDC’s motor function level. Particularly notable was that as they became mobile, TDC removed devices by themselves, and similar problems were described in the process of care [21]. Therefore, overall, the improvement of motor function does not ease caregivers’ burden as had been previously thought. Rather, increased mobility often forces caregivers to pay constant attention to the child. In Japan, the social welfare system covers daycare services, home helpers, and other living expenses. However, “the social welfare system for *children with severe motor and intellectual disabilities* (C-SMID)” is limited only to immobile TDC. As they become mobile, they become ineligible to receive such support, causing a serious problem for TDC and caregivers.

In the follow-up group, nearly half of TDC (106/262) required the support of 3 devices (tracheostomy+ventilator+gastrostomy tube), and their conditions seem severe. Yet, it is noteworthy that even in this group, 4 children were freed from 1 or 2 devices, and in one case, all 3 devices were removed after 4 years (Fig. 4). Becoming mobile gives a higher care burden, but it also gives caregivers hope for recovery. Therefore maintaining social support for TDC of the mobile stage will also help their withdrawal from devices.

Japan has 10 years of experience in implementing the PHMC for TDC and experienced problems that have arisen along with TDC’s growth. Well-coordinated home care systems have been proposed and implemented in many locations [22, 23]. Still, as the number of TDC is rapidly increasing worldwide, many nations would face similar problems such as those described in this paper.

## Conclusion

In their recovery process of motor function, TDC cause more trouble for caregivers, and providing appropriate public support for this group of TDC could decrease the heavy burden on their families, and facilitate TDC’s recovery from conditions in need of multiple devices. Future problems are the steady increase of TDC who need PHMC, and providing PHMC services for all of these children may require more clinics to join the system. Furthermore, as the number of children who leave PHMC increases, additional clinical outpatient systems will need to be developed for their after-care as well.

### Supplementary Information

Below is the link to the electronic supplementary material.

Supplementary file1 (DOCX 24 KB)

## Data Availability

All data generated or analyzed during this study are included in this published article.

## Abbreviations

CSMID: Children with severe motor and intellectual disabilities
CT: Care time
IV: Invasive ventilation
NIV: Non-invasive ventilation
SCB: Self-rated care burden
PHMC: Pediatric home medical care
TDC: Technology-dependent children

## Declarations

### Disclaimer﻿

The Japanese Ministry of Health, Welfare, and Labor had no role in the design and conduct of the study.
