# Supplementary material for: The care burden for technology-dependent children with long-term home ventilation increases along with the improvement of their motor functions
Source: Eur J Pediatr. 2023 Oct 16;183(1):135–47. doi: 10.1007/s00431-023-05249-w (PMC10858118; doi:10.1007/s00431-023-05249-w)
Supplement: Supplementary file 1 — Supplementary file1 (DOCX 24 KB) [file 431_2023_5249_MOESM1_ESM.docx]

**Supplementary information**

**Supplementary Table 1:** List of disease names in each category

|  | Supplementary Table 1: List of disease names in each category |  |
| --- | --- | --- |
|  | **Chromosomal Abnormality** |  |
|  | 10p syndrome |  |
|  | Trisomy 13 |  |
|  | Trisomy 18 |  |
|  | 1p35.3 15q11 1q13.1 deletion |  |
|  | Trisomy 21 |  |
|  | 22q.11.2 deletion syndrome |  |
|  | Chromosome 3 long arm partial trisomy |  |
|  | 4p deletion syndrome |  |
|  | Chromosome 6 long arm partial deletion |  |
|  | Tetrasomy 12p (Pallister-Killian syndrome) |  |
|  | Autosomal monosomy 46, XY, add(7)(p22) |  |
|  | Chromosome abnormality G-band 46 XX t4:10 q21;p11.2 |  |
|  | Monosomy 14 |  |
|  | **Internal Organ Disease** |  |
|  | ASD VSD |  |
|  | DORV MA Coac HLHS |  |
|  | Hirschsprung's disease |  |
|  | TOF |  |
|  | Dilated cardiomyopathy |  |
|  | Complete transposition of great arteries |  |
|  | Laryngomalacia |  |
|  | Tricuspid valve closure, aortic coarctation |  |
|  | Esophageal atresia |  |
|  | Progressive familial intrahepatic cholestasis type 1 |  |
|  | Glottal stenosis |  |
|  | Congenital lymphangiogenesis disorder |  |
|  | Tracheal stenosis |  |
|  | Anomalous common pulmonary venous return |  |
|  | Aortic transection type B, ventricular septal defect, aortic stenosis |  |
|  | Short bowel syndrome |  |
|  | Infantile dural arteriovenous fistula |  |
|  | Pulmonary artery atresia |  |
|  | Adrenoleukodystrophy |  |
|  | Atrioventricular septal defect |  |
|  | Chronic thrombocytopenic purpura |  |
|  | **Metabolic Disease** |  |
|  | Gaucher disease (type 3)（type２） |  |
|  | GM3 synthase deficiency |  |
|  | Leigh encephalopathy |  |
|  | Ornithine transcarbamylase deficiency |  |
|  | Niemann-Pick type C |  |
|  | Pyruvate dehydrogenase complex deficiency |  |
|  | Metachromatic leukodystrophy |  |
|  | Perinatal severe hypophosphatasia |  |
|  | Non-ketotic hyperglycinemia |  |
|  | **Multiple Congenital Malformation Syndrome** |  |
|  | Unclassified Chromosome abnormality |  |
|  | CFC-syndrome (Cardio-facio-cutaneous syndrome) |  |
|  | CHARGE syndrome |  |
|  | Costello syndrome |  |
|  | Emanuel syndrome |  |
|  | Joubert syndrome |  |
|  | Marshall-Smith syndrome |  |
|  | RETT syndrome |  |
|  | Smith-Kingsmore syndrome |  |
|  | VACTERL association |  |
|  | Apert syndrome |  |
|  | Goldenhar syndrome |  |
|  | Cornelia de Lange syndrome |  |
|  | Sotos syndrome |  |
|  | Dundee-Walker syndrome |  |
|  | Pierre Robin syndrome |  |
|  | Pfeiffer syndrome type 2 |  |
|  | Mitochondrial disease |  |
|  | Malformation syndrome |  |
|  | Tracheobronchomalacia |  |
|  | Thoracic hypoplasia, pulmonary hypoplasia |  |
|  | Campomelic dysplasia |  |
|  | Posterior cranial fossa lump |  |
|  | Geleophysic Dysplasia |  |
|  | Skeletal dysplasia |  |
|  | Osteocraniostenosis |  |
|  | Severe combined immunodeficiency |  |
|  | Congenital tracheal stenosis |  |
|  | Congenital osteogenesis imperfecta type III |  |
|  | Arthroglyposis multiplex congenita(AMC) |  |
|  | Holoprosencephaly |  |
|  | Premature very low birth weight child (28w, BBW 1140g) |  |
|  | Punctate cartilage anomaly(chondrodysplasia punctata) |  |
|  | Metatropic dysplasia |  |
|  | **Neuromuscular Disease** |  |
|  | SMA1 |  |
|  | VICI syndrome |  |
|  | Athetotic cerebral palsy |  |
|  | West syndrome |  |
|  | Epilepsy |  |
|  | Myotubular myopathy |  |
|  | Psychomotor retardation |  |
|  | Open spinal meningioma |  |
|  | lissencephaly |  |
|  | Miller-Deiker syndrome |  |
|  | Autoimmune encephalitis |  |
|  | Syringomyelia |  |
|  | Spinocerebellar degeneration |  |
|  | Myelomeningocele |  |
|  | Spinal muscular atrophy type I |  |
|  | Congenital myopathy |  |
|  | Congenital myotonic dystrophy |  |
|  | Early epileptic encephalopathy |  |
|  | Ohtahara Syndrome |  |
|  | Cerebral palsy |  |
|  | muscular dystrophy |  |
|  | Encephalopathy |  |
|  | **Peri/Postnatal Disorder** |  |
|  | Influenza encephalopathy |  |
|  | Viral encephalopathy sequela |  |
|  | Ischemic encephalopathy after cardiac arrest |  |
|  | Steroid-resistant ulcerative colitis |  |
|  | Bronchial foreign body |  |
|  | Acute flaccid myelitis sequela coxsackie virus |  |
|  | Acute encephalopathy sequela |  |
|  | Neonatal cerebral hemorrhage sequelae |  |
|  | Neonatal hypoxic-ischemic encephalopathy |  |
|  | Meningitis/septicemia |  |
|  | Congenital cytomegalovirus infection |  |
|  | Twin-to-twin transfusion syndrome |  |
|  | Premature low birth weight |  |
|  | Head injury (Acute subdural hematoma) |  |
|  | Post-traumatic head injury |  |
|  | **Tumor** |  |
|  | Lymphangioma |  |
|  | Rhabdomyosarcoma |  |
|  | Acute lymphocytic leukemia |  |
|  | Osteosarcoma |  |
|  | Cerebellar medulloblastoma |  |
|  | Myelomeningocele |  |
|  | Atypical teratoid/rhabdoid tumor |  |
|  |  |  |
